# Circulating Myokines as Novel Biomarkers for Cardiovascular Diseases

**DOI:** 10.31083/j.rcm2502056

**Published:** 2024-02-05

**Authors:** Jin-xiu Lyu, Dan-dan Guo, Yu-chen Song, Man-ru Zhang, Feng-qin Ge, Jing Zhao, Hua Zhu, Peng-zhou Hang

**Affiliations:** ^1^Department of Pharmacy, Clinical Medical College, Yangzhou University, Northern Jiangsu People's Hospital, 225001 Yangzhou, Jiangsu, China; ^2^Medical College, Yangzhou University, 225009 Yangzhou, Jiangsu, China; ^3^College of Pharmacy, Dalian Medical University, 116044 Dalian, Liaoning, China

**Keywords:** myokine, cardiovascular diseases, biomarker

## Abstract

Myokines are a group of cytokines or polypeptides released from skeletal muscle during exercise. Growing evidence suggests that myokines are associated with the development of cardiovascular disease (CVD). Moreover, several myokines in peripheral blood exhibit dynamic changes in different CVD stages. This review summarizes the potential roles of myokines such as myostatin, irisin, brain-derived neurotrophic factor, mitsugumin 53, meteorin-like, and apelin in various CVD, including myocardial infarction, heart failure, atherosclerosis, hypertension, and diabetes. The association of these myokines with biomarkers currently being used in clinical practice is also discussed. Furthermore, the review considers the emerging role of myokines in CVD and addresses the challenges remaining in translating these discoveries into novel clinical biomarkers for CVD.

## 1. Introduction

**Fig. 1.**
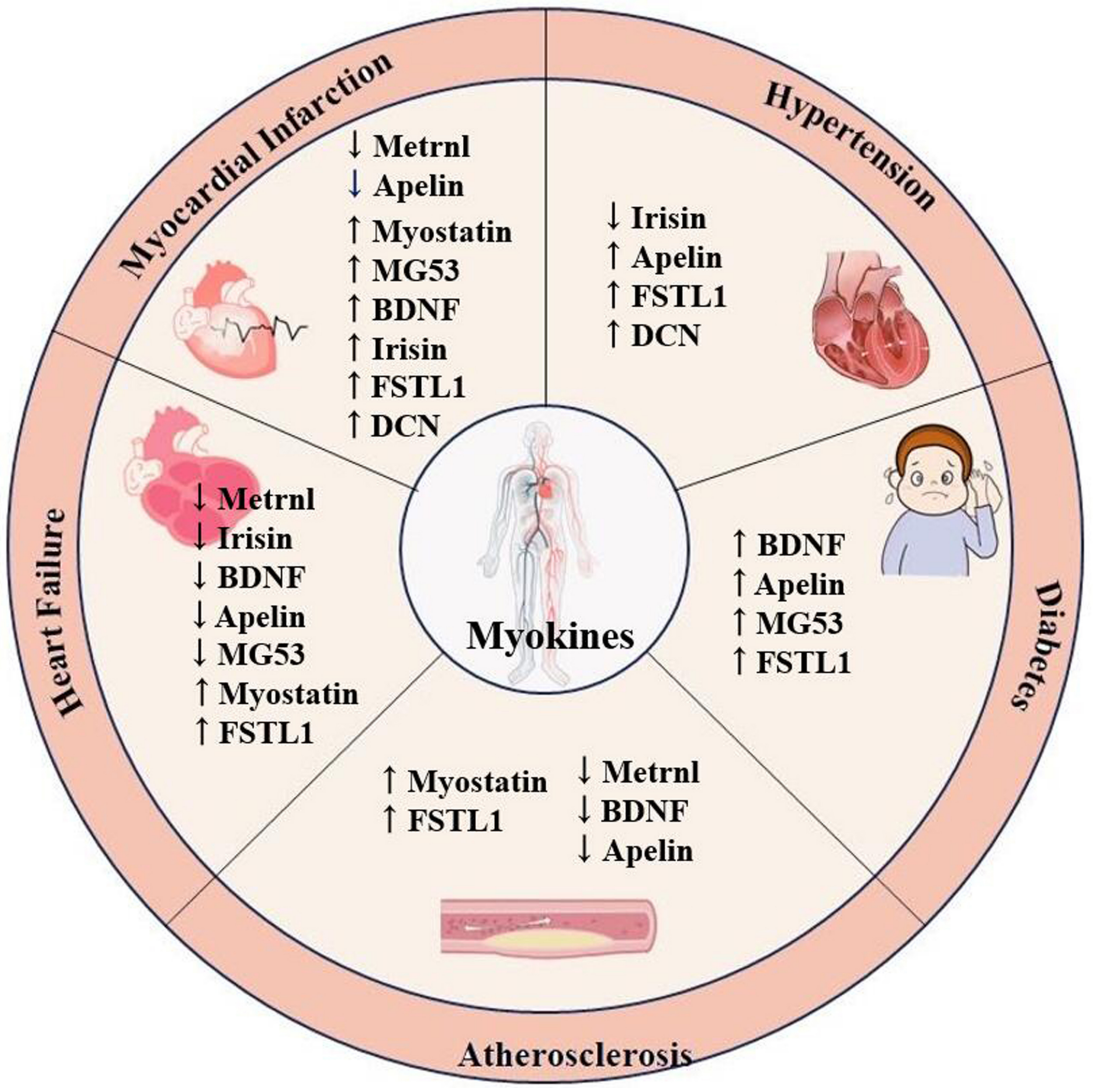
**Association between circulating myokines and cardiovascular diseases including myocardial infarction, heart failure, atherosclerosis, diabetes, and hypertension**. Metrnl, meteorin-like; MG53, mitsugumin 53; BDNF, brain-derived neurotrophic factor; FSTL1, follistatin-like 1; DCN, decorin.

The term “myokines” was coined in 2003 referring to a group of substances known as “exercise factors” released into the bloodstream by various tissues during exercise [1]. These cytokines are minuscule proteins (5–20 kDa) and proteoglycan peptides generated, expressed, and discharged by muscle fibers in response to contraction. They have a localized autocrine and paracrine impact, and have a distant endocrine influence on different organs including the heart [2]. Skeletal muscle interacts with metabolic systems, such as adipose tissue, the liver, and the pancreas, by releasing endocrine factors, including myokines [3]. Myokines can modulate adipose tissue metabolism and thermogenic activity [4]. Other myokines identified potentially played a pivotal role in obesity management and its correlated cardiac complications [5]. The discovery of novel pathways and the link between myokines and cardiovascular disease (CVD) is essential for developing comprehensive treatments [6]. Several myokines, including myostatin, irisin, brain-derived neurotrophic factor (BDNF), mitsugumin 53 (MG53), meteorin-like (Metrnl), apelin (AP), follistatin-like 1 (FSTL1), decorin (DCN), and myogenin, participate in regulating the pathogenesis of CVD (Fig. 1). Measuring these myokines in peripheral blood offers novel perspectives on CVD advancements and enables clinicians to stratify patients by their risk [7].

## 2. Myostatin

Myostatin, referred to as growth differentiation factor 8 (GDF-8), belongs to the transforming growth factor-β (TGF-β) superfamily. It is a crucial player in the negative regulation of myogenesis through autocrine or paracrine signaling [8]. This secretory polypeptide mediates cellular communication. The myostatin gene factor is crucial for the postnatal downregulation of muscle fiber size and quantity. Myostatin knockout mice have a greater myofiber size, muscle weight, and grip strength than their heterozygous and wild-type littermates [9]. A growing body of evidence indicates that high myostatin levels in the bloodstream are correlated with an elevated CVD risk as documented below.

### 2.1 Myostatin in Myocardial Infarction

Reducing myostatin expression by inhibiting activin type II receptor (ACTRII) can inhibit pathological cardiac remodeling in ischemic cardiomyocytes [10]. Furthermore, myostatin expression in vascular smooth muscle cells (VSMCs) can impede the function of 14q32 microRNAs, thereby inhibiting both VSMCs proliferation and local inflammation. However, it does not at restenosis after intervention [11]. Furthermore, serum myostatin levels were indicative of myocardial damage severity during acute myocardial infarction (AMI), akin to the currently used troponin I peak that estimates infarct size [12]. A prospective observational study revealed a correlation between lower serum myostatin levels and higher mortality rates among patients hospitalized for myocardial infarction (MI) [13]. Moreover, in that study, the myostatin concentration was positively linked to muscle mass and strength in patients with ST-segment elevation MI (STEMI) [13]. Meanwhile, multiple studies have reported that myostatin levels increase in the hearts and blood of mice 12 h after MI and 10 min after ischemia. Myostatin was also induced in rat kidney ischemia and reperfusion conditions and was correlated with mitochondrial damage [14]. These studies thus suggest that myostatin is involved in MI-related cachexia development, and its expression seems upregulated during MI, indicating its potential as a valuable clinical marker.

### 2.2 Myostatin in Heart Failure

Myostatin expression in skeletal muscle is higher in patients with heart failure (HF) having a reduced ejection fraction (HFrEF) compared to healthy controls. It is also higher in HF patients with a preserved ejection fraction (HFpEF) [15]. Furthermore, myostatin deficiency reduces myocardial interstitial fibrosis and protects cardiac function [16]. Additionally, the expression of myostatin and its receptors is higher in the left ventricles of patients with advanced HF than in the left ventricles of healthy subjects [17]. By contrast, lower serum myostatin levels were observed in patients with HF, which may be linked to lower limb muscle atrophy [18]. Moreover, on investigating the correlation between serum myostatin levels and both disease severity and prognosis in patients with congestive HF, Chen *et al*. [19] discovered that myostatin levels were higher in patients with congestive HF than in the control group. Additionally, Cox regression analysis demonstrated that serum myostatin is an independent predictor of mortality. These studies thus provide evidence that myostatin expression is significantly upregulated in HF patients. Nevertheless, whether myostatin can be used as a clinical marker for HF needs to be validated further.

### 2.3 Myostatin in Atherosclerosis

Early studies have reported that serum myostatin regulates skeletal muscle growth and extracellular matrix production. Furthermore, Serum myostatin levels are independently associated with increased aortic stiffness in adolescents. These findings suggest that muscular factors contribute to the early onset of systemic hypertension and vascular aging [20]. Myostatin in the vascular wall is essential for arterial aging and aortic atherosclerosis (AS) progression. Myostatin expression has been observed in neointima, new vessels, and infiltrating cells at atherosclerotic lesion sites, with the levels increasing with the progression of vascular injury [21]. Collectively, these studies propose that myostatin can play a role in the fundamental mechanisms contributing to vascular aging and related illnesses and that it offers a theoretical basis for developing therapeutic interventions targeted at AS and vascular calcification [22].

### 2.4 Myostatin in Other CVD

Myostatin expression decreases in the hearts of patients with persistent atrial fibrillation. Moreover, *in vitro* experiments conducted using a synthetic peptide corresponding to a distinct DCN region demonstrated a significant dose-dependent reduction in the response to myostatin in cardiomyocytes and perfused mouse hearts [23]. Furthermore, myostatin levels have been reported in cats with congestive HF and hypertrophic cardiomyopathy. The plasma samples were subjected to liquid chromatography-tandem mass spectrometry to determine the myostatin levels. The cats with hypertrophic cardiomyopathy or hypertrophic obstructive cardiomyopathy exhibited higher myostatin levels than those with congestive HF [24].

In a Japan-based cross-sectional study measuring serum myostatin levels in obese hyperinsulinemic patients, the myostatin levels were positively correlated with serum immunoreactive insulin levels [25]. This indicated the potential pathological significance of myostatin in muscle mass and metabolism in obese hyperinsulinemic patients [25]. Furthermore, antihypertensive and lipid-lowering medications decrease myostatin levels, which is a plasma protein biomarker for CVD [26].

## 3. Irisin

Irisin, a 112-amino acid-long myokine dependent on peroxisome proliferator-activated receptor gamma coactivator 1 α (PGC1α), was initially believed to be an exercise-induced molecule with beneficial effects [27]. Fibronectin type III domain-containing protein 5 serves as the precursor protein that is proteolytically cleaved to produce irisin, which is secreted by skeletal muscle cells and cardiomyocytes [28]. Recent studies have highlighted the potential utility of irisin as a biomarker for CVD diagnosis [29]. Irisin, produced by skeletal and cardiac muscles, has a considerable impact on various cardiovascular functions. For instance, during the early AMI stages, elevated irisin levels may impede inflammation and oxidative stress and decrease endothelial damage. However, these elevated levels during the later MI stages are linked to a higher incidence of cardiovascular events [30].

### 3.1 Irisin in MI

The serum irisin levels are increased in patients with myocardial ischemia/reperfusion (I/R) injury, which may be released from injured cardiomyocytes. Intravenously injected exogenous irisin offers dose-dependent shielding against I/R injury, which is achieved by augmenting the levels of superoxide dismutase-1 (SOD-1) [31]. This injection also increases the levels of antioxidant enzymes, including glutathione, SOD, and glutathione peroxidase, thereby decreasing reactive oxygen species levels [32]. Irisin was recently found to modulate the endoplasmic reticulum–mitochondria interaction through the mitochondrial ubiquitin ligase-dependent pathway, which reduces myocardial I/R-induced heart dysfunction [33].

Studies involving an isoproterenol-induced rat MI model have indicated that irisin negatively correlates with troponin and creatine phosphokinase-myocardial zone isoenzyme (markers of MI). These findings suggest that irisin is secreted as a protective factor for injured cardiomyocytes [34]. In addition, irisin has the potential to act as an anti-I/R agent by regulating myocardial cell death. Irisin treatment significantly reduces active caspase-3 production in cardiomyocytes that have undergone hypoxia and reoxygenation [35]. Furthermore, *in vitro* experiments have revealed that irisin promotes the proliferation and angiogenesis of human umbilical vein endothelial cells (ECs) through the ERK signaling pathway. In addition, irisin activates the Akt/mTOR/Nrf2 pathway, thereby mitigating oxidized low-density lipoprotein (LDL)-induced angiogenic damage [36].

### 3.2 Irisin in HF

Irisin acts as a pro-myogenic factor, and its serum expression significantly increases after exercise. According to preclinical studies, irisin exerts beneficial effects on the heart by promoting cardiac remodeling, enhancing cardiomyocyte viability and calcium delivery, and reducing inflammatory mediators, thereby providing cardioprotective effects. Therefore, irisin has a significant impact on HF prognosis [37]. Decreased serum irisin levels were reported in patients with sarcopenia, combined with chronic obstructive pulmonary disease (COPD) and chronic heart failure (CHF) [38]. Additionally, other studies have shown that HFrEF patients with cardiac cachexia had considerably greater serum adropin and irisin levels than the controls [39].

Furthermore, serum irisin levels were lower in HFrEF patients than in healthy volunteers but were significantly higher in HFpEF patients. This suggested that irisin levels serve as an indicator of multi-system disease in HFpEF patients [40]. Interestingly, decreased serum irisin levels were observed to be a significant predictor of HFpEF patients with type 2 diabetes mellitus (T2DM), but not in HF patients with mid-range ejection fraction (HfmrEF) and HFrEF, which can potentially make way for a new approach for stratifying HF risk among T2DM patients [41]. Similarly, serum irisin levels can serve as predictors of cumulative clinical outcomes in individuals with both HF and T2DM [42]. These investigations indicate that irisin has the potential as a reliable indicator of cardiovascular system efficacy and systemic metabolism in CHF patients.

### 3.3 Irisin in AS

ECs are crucial for AS development, as they need to be preserved for maintaining vascular homeostasis and acting as a selective osmotic barrier between circulating blood and surrounding tissues. Irisin has recently emerged as a promising agent for improving endothelial function in CVD treatment. Oxidative LDL induces EC apoptosis, which can be effectively reduced through irisin treatment. Furthermore, systemically administered irisin hampers AS by activating the AMP-activated protein kinase-phosphatidylinositide 3-kinases-Akt-endothelial nitric oxide synthase (AMPK-PI3K-Akt-eNOS) signaling pathway [43]. Nonetheless, whether irisin can modify the VSMCs phenotype and thus exert an anti-atherosclerotic effect requires to be validated in future studies.

### 3.4 Irisin in Hypertension and Diabetes

Arterial hypertension (AH), diabetes mellitus (DM), and chronic kidney disease (CKD) are prevalent risk factors for cardiovascular events. Irisin can reduce blood pressure through a nitric oxide (NO)-dependent pathway. In women with pre-eclampsia, irisin levels are inversely related to blood pressure values. Irisin also plays a critical role in regulating insulin sensitivity and mitigating metabolic disorders. Irisin levels were lower in T2DM patients than in non-diabetic controls. Furthermore, serum irisin levels were reduced in pregnant women with gestational DM (GDM) [44]. Studies have reported on the expression of irisin and urotensin II (UII) and their association with blood pressure in patients with pre-eclampsia. Serum irisin was negatively correlated with both systolic and diastolic blood pressure, while serum UII was positively correlated with systolic blood pressure in pre-eclampsia patients. Additionally, a multiple regression analysis revealed that serum irisin, serum UII, urine protein, body mass index (BMI), and serum creatinine (Cr) are independent determinants of blood pressure in pre-eclampsia [45]. The study investigated the potential association between plasma irisin levels and hemodynamic dysfunction in patients with idiopathic pulmonary arterial hypertension (IPAH) and assessed whether irisin serves as a predictor of clinical outcome. The findings revealed that plasma irisin levels were positively correlated with total cholesterol (TC) and LDL cholesterol (LDL-C) levels in the high irisin group. By contrast, IPAH patients with low irisin levels had significantly increased arterial pressures, which were negatively correlated with the irisin levels. Notably, plasma irisin levels can independently predict prognosis in IPAH patients [46]. In a cross-sectional study, hormonal and metabolic parameters were evaluated, alongside the carotid intima-media thickness (cIMT) and epicardial fat thickness (EFT) measured through an ELISA. The study indicated that study patients with acromegaly had considerably elevated circulating irisin levels compared with the control group. Moreover, patients with controlled acromegaly exhibited even higher irisin levels. Remarkably, irisin correlates positively with insulin resistance, cIMT, EFT, BMI, growth hormone, and insulin-like growth factor [47].

Irisin exerts a protective effect against diabetic cardiomyopathy through various mechanisms. First, it activates integrin αV/β2-AKT signaling, thereby reducing oxidative/nitrosating stress and alleviating diabetic cardiomyopathy in type 2 diabetic mice models [48]. Second, irisin reduces blood pressure in diabetic rats by inhibiting the NF-κB signaling pathway [49]. Additionally, it can improve high glucose-induced cardiomyocyte damage through the AMPK/mTOR signaling pathway [50].

## 4. Brain-Derived Neurotrophic Factor

BDNF, which is widely expressed in the adult brain, is a crucial player in both neurogenesis and neuroplasticity. BDNF, produced by the muscle itself, remodels the neuromuscular synapse, the neuronal connections between motor neurons and muscles [51]. BDNF hypermethylation is linked to an increased CVD risk, which may be useful for identifying individuals at CVD risk. Additionally, BDNF hypermethylation was positively correlated with CVD severity [52].

### 4.1 BDNF in MI

The association between serum BDNF levels and MI has been well-documented. Mounting evidence has indicated the protective effect of BDNF on I/R injury. Intravenous injected BDNF inhibits cell death and protects against myocardial I/R injury. This protective effect is achieved by suppressing mitochondrial superoxide anion production and activating the BDNF/TrkB axis [53]. Collectively, the BDNF level is closely associated with MI, and the serum BDNF level can serve as a predictive marker of cardiovascular events. For instance, BDNF accumulation predicted the cardiovascular outcome following glucose intake, where the low BDNF group exhibited a significantly higher risk of end outcomes than the high BDNF group [54]. A comparative study investigated the association between BDNF and the renin enzyme before and after percutaneous coronary intervention (PCI), and the role of this enzyme in patients with coronary heart disease (CHD). By measuring serum BDNF and renin enzyme levels before and after PCI, the survival rate of CHD patients can be predicted. The findings revealed that the percentage change in serum BDNF levels before PCI was inversely correlated with that in renin enzyme levels after PCI [55].

### 4.2 BDNF in HF

Numerous studies have indicated that serum BDNF levels and the prediction, diagnosis, and prognosis of HF are closely related. Specifically, miR-182-5p combined with BDNF is known to aid CHF diagnosis. In individuals with CHF, serum miR-182-5p was positively correlated with B-type natriuretic peptide (BNP), whereas miR-182-5p was negatively correlated with left ventricular ejection fraction (LVEF). Additionally, serum BDNF was negatively linked with BNP and positively linked with LVEF. Consequently, miR-182-5p combined with BDNF can be used for effectively diagnosing CHF, as well as for predicting an unfavorable prognosis [56]. Additionally, growth-stimulating expression gene 2 protein, cardiac troponin I, blood urea nitrogen, and Cr were expressed at high levels in the serum of HF patients, whereas BDNF was expressed at low levels [57]. The combination of serum BDNF levels and peak ${\mathrm{VO}}_{2}$ at discharge can serve as helpful predictors for early cardiac events [58]. The reduced serum BDNF level is linked to patient mortality and re-hospitalization, which underscores its value as a prognostic biomarker [59]. Decreased serum BDNF levels in patients with Chagas cardiomyopathy, especially when associated with systolic function, may also offer useful prognostic information [60]. Intriguingly, computerized cognitive training can improve memory and reduce serum BDNF levels in HF patients [61].

In general, patients with CHF and stroke have lower BDNF levels, whereas those with unstable angina and recent MI have higher BDNF levels. Moreover, reduced BDNF levels were linked to increased occurrence of cardiovascular events among patients with a history of severe CVD, whereas a lower cardiovascular risk was observed among healthy individuals. As a critical biomarker for HF, BDNF needs to be further explored [62].

### 4.3 BDNF in AS

BDNF may mitigate DM-induced AS (DMAS). To examine this, BDNF levels in the serum and peripheral blood mononuclear cells (PBMCs) were assessed in DMAS patients and healthy controls through real-time quantitative PCR and western blotting. The expression of inflammatory cytokines (IL-1β, TNF-α, IL-10, TGF-β, and IL-13) was also assessed. How BDNF repair affects cytokine release, macrophage differentiation, and atherosclerotic plaque formation was evaluated *in vitro* and *in vivo* by using a mouse model of DMAS. Lower BDNF levels were found in the serum and PBMCs of DMAS patients. Furthermore, BDNF overexpression prompted M2 macrophage polarization. Inhibiting the signal transducer and activator of transcription 3 (STAT3) pathway impeded DMAS progress [63]. Moreover, the expression and signal transduction of BDNF have been investigated in varied perivascular adipose tissues among CHD patients. Subsequently, serum, vascular tissue, and perivascular adipose tissue samples close to the proximal aorta (C-PVAT) or internal mammary artery (IMA-PVAT) were collected. BDNF protein levels were significantly higher in the C-PVAT group than in the IMA-PVAT group. These findings suggest that BDNF can serve as an autonomous biomarker, irrespective of obesity, metabolic syndrome, or systemic inflammation [64].

### 4.4 BDNF in Diabetes

BDNF plays a crucial role in pre-diabetes and T2DM cognitive impairment, where serum levels are considerably increased in T2DM patients. BDNF, therefore, could be a biomarker affected by T2DM and cognition [65]. The association between miR-1-3p expression and BDNF levels has been investigated in patients with essential hypertension. Both RT-qPCR and ELISA analyses demonstrated that miR-1-3p expression significantly increased, whereas serum BDNF levels decreased in these patients. Furthermore, miR-1-3p was negatively correlated with BDNF and acted by negatively regulating its expression [66]. Meanwhile, infants of mothers with GDM had significantly lower serum BDNF levels than the controls [67]. Notably, BDNF has been linked to microcirculation ischemia, reflecting myocardial cell damage. A study in South Africa explored the expected changes in BDNF and cardiac troponin T (cTnT), with elevated BDNF levels leading to chronically lower cTnT levels [68].

## 5. Mitsugumin 53

MG53, also referred to as TRIM72, belongs to the tripartite motif protein (TRIM) family. It comprises a conventional TRIM domain consisting of RING, B-box, and coiled-coil domains at its N-terminal end and a SPRY domain at its C-terminal end. MG53 plays a dual role in the heart: on the one hand, it is involved in repairing the cell membrane that protects myocardial function. on the other hand, it acts as an E3 ligase that triggers insulin resistance and cardiovascular complications [69]. Therefore, emerging research indicates that MG53 levels can be used as a predictive indicator and a prognostic and diagnostic marker for CVD.

### 5.1 MG53 in MI

MG53 is an E3 ubiquitin ligase that swiftly accumulates at the membrane damage site and plays a critical role in repairing membranes of skeletal and cardiac muscles. MG53 E3 ligase-death mutants have been reported to protect diabetic hearts from I/R injury and ameliorate diet-induced cardiometabolic injury [70]. Treatment with recombinant human MG53 (rhMG53) protected cardiac function from I/R-induced oxidative stress by safeguarding the mitochondrial function in cardiomyocytes [71]. Moreover, in a study evaluating the MG53 prognostic value in STEMI patients, serum MG53 levels were measured in 300 patients followed up for 3 years. MG53 was noted to be an effective prognostic marker for major adverse cardiovascular events in AMI patients, independent of established traditional risk factors [72]. MG53 levels were elevated in patients with stable CHD and those with comorbidities such as CKD and DM, and highest in those with AMI. Furthermore, the severity of CVD and AMI correlated with MG53 levels after adjustment for multiple risk factors and clinical biomarkers [73]. The use of MG53 as a clinical marker of MI is controversial, and further studies are warranted to fully understand the potential utility of MG53 in this context.

### 5.2 MG53 in HF

Both the impairment of myocardial cell integrity and aging are underlying factors of HF in humans. MG53 expression decreases in both failing human hearts and aging mice hearts, concomitant with heightened NF-κB activation. Notably, recombinant human MG53 improves cardiac function when administered to elderly mice, as evidenced by echocardiography and pressure-volume loop measurements [74]. MG53 exerts a dual effect on the heart: it participates in repairing cell membranes to safeguard myocardial function, while simultaneously serving as an E3 ligase that elicits insulin resistance and cardiovascular complications [75].

### 5.3 MG53 in AS

Vascular ECs play a prominent role in AS initiation and progression. According to recent studies, MG53 remarkably contributes to the modulation of EC function. To clarify this phenomenon, researchers have applied rhMG53 to human umbilical vein ECs *in vitro* and evaluated uptake, activation of the adhesion spot kinase focal adhesion kinase/Src/Akt/extrallular signal regulated protein kinase1/2 (FAK/Src/Akt/ERK1/2) signaling pathway, and cell migration and tubule formation. The outcomes revealed that rhMG53 significantly inhibited angiogenesis by modifying the FAK/Src/Akt/ERK1/2 signaling pathway, thereby suggesting a novel molecular mechanism for impaired angiogenesis in ischemic disorders [76].

### 5.4 MG53 in Diabetes

Metabolic syndrome, characterized by obesity, insulin resistance, and hyperlipidemia, is linked to an increased CVD risk. A prospective study evaluated the relationship between MG53 and glucose tolerance, with circulating levels tested in a high-risk cohort of T2DM patients to assess disease progression. The study indicated that patients with impaired glucose regulation or T2DM had significantly higher MG53 levels than those with normal glucose tolerance (NGT) [77].

To better understand the potential role of MG53, studies have examined its activity in a mouse model of metabolic disorders induced by feeding a high-fat diet for 6 months. They reported that MG53 expression remained unchanged in the skeletal muscle and myocardium of metabolic syndrome mice; however, circulating MG53 levels were downregulated [78]. The results suggest that therapeutic intervention aimed at addressing the interaction between MG53 and insulin receptor substrate 1 (IRS-1) can be developed, which could serve as a novel strategy for treating insulin resistance-linked metabolic conditions [79].

## 6. Meteorin-Like

Meteorin-like (Metrnl) is a recently discovered secretory protein that activates several intracellular signaling pathways in different cell types, including adipocytes, macrophages, myocytes, and cardiomyocytes. This activation triggers various physiological effects, including browning of the white adipose tissue, increased insulin sensitivity, inhibition of inflammation, skeletal muscle regeneration, and protection of the heart [80].

Several studies have recently investigated the involvement of Metrnl in the development of coronary artery disease (CAD). Liu *et al*. [81] examined the association between serum Metrnl protein and CHD in Chinese adults. ELISAs were conducted to determine serum Metrnl levels. An adverse correlation was observed between serum Metrnl levels and metabolic parameters, such as BMI, TC, LDL-C, and inflammatory indicators. Furthermore, a reduction in Metrnl levels was negatively correlated with CVD severity, as assessed by the Gensini score. This indicated that Metrnl may serve as a promising new biomarker for CHD. Similarly, decreased serum Metrnl levels were noted in AMI patients and were negatively correlated with the time of onset [82]. Additionally, Cai and colleagues [83] demonstrated that CHF patients had significantly lower serum Metrnl levels than the control group. Moreover, serum Metrnl levels were inversely correlated with LVEF. Furthermore, decreased serum Metrnl levels are associated with impaired glucose tolerance, compromised endothelial function, and AS. Metrnl serves as a suitable surrogate marker for endothelial dysfunction and AS and as an independent risk factor for T2DM since it allows the evaluation of insulin resistance [84].

Metabolic syndrome, T2DM, and CAD exhibit an association with Metrnl. In a meta-analysis on T2DM patients and healthy controls, no significant association was observed between serum Metrnl levels and the risks of T2DM and CAD. However, serum Metrnl levels were significantly associated with obesity. Further verification is therefore required to establish the relationship between Metrnl and T2DM and CAD [85]. As a myokine, Metrnl plays a crucial role in metabolic syndrome. It has cardioprotective properties when found in blood and serves as a cardiac factor in heart disease. Additionally, Metrnl can potentially serve as a therapeutic target for inflammatory myopathies and aging [86].

Studies have evaluated the impact of maternal obesity and GDM on Metrnl levels in cord and plasma. Plasma Metrnl levels were significantly lower in non-GDM obese women than in non-obese women with NGT as well as in non-obese women with GDM than in non-obese women with NGT. Metrnl levels were positively correlated between maternal and cord plasma. In cord plasma, significant positive links were observed between Metrnl levels and gestational weight gain, as well as between Metrnl levels and maternal and cord plasma glucose levels during delivery [87]. In a study [88], the diabetic group exhibited significantly lower serum Metrnl levels than the control group. Conversely, serum asprosin levels were notably higher in the diabetes group than in the control group. Furthermore, asprosin levels were positively associated with homeostasis model assessment–insulin resistance (HOMA-IR), insulin, BMI, and triglyceride (TG) levels in the patient group. By contrast, Metrnl levels were inversely correlated with HOMA-IR, insulin, TG levels, and glucose levels [88]. Furthermore, in T2DM patients, Metrnl levels increased for the first time and were inversely correlated with several cardiometabolic risk factors, including renal function [89]. To assess serum Metrnl levels and their impact on glucose and lipid metabolism among obese adult patients, serum Metrnl levels were measured using an ELISA. Serum Metrnl levels were notably lower in overweight or obese individuals than in the normal group. Additionally, circulating Metrnl levels were negatively correlated with TG, TC, LDL-C, and small dense low-density lipoprotein and positively correlated with high-density lipoprotein cholesterol (HDL-C), both before and after adjustments for age, sex, BMI, diabetes, HOMA-IR, and estimated glomerular filtration rate (eGFR) [90]. A study investigated the association between serum Metrnl levels and visceral adiposity (VFO) in Chinese patients with T2DM [91]. In that study, the VFO group displayed lower serum Metrnl levels than the non-VFO group. Correlation analyses indicated that serum Metrnl levels were negatively associated with visceral fat accumulation, TC, TG, LDL-C, and albumin, but were positively associated with age, height, BUN, Cr, and uric acid [91].

## 7. Apelin

AP was first discovered in 1993 as a G-protein-coupled receptor. Being approximately 50% similar to the angiotensin type 1 (AT1) receptor, AP inhibits angiotensin II (Ang II) agonists on AT1 receptors both *in vitro* and *in vivo * [92]. AP and AT1 receptors have been found in the cardiovascular system. The 77-amino acid precursor of AP gives rise to the primary isomers AP-36, AP-17, and AP-13, of which AP-13 is the most prevalent subtype in the cardiovascular system and human plasma. Furthermore, the AP receptor system plays a crucial role in regulating cardiovascular physiology and pathology, which highlights its significance as a potential target for cardiovascular drug discovery and development [93].

### 7.1 Apelin in MI

MI is a significant cause of cardiovascular morbidity and mortality. AP, a hormone produced by the heart, is crucial for maintaining heart health and exerts atheroprotective, antihypertensive, and regenerative effects [94]. Liu *et al*. [95] reported that serum AP levels can forecast major adverse cardiac events in STEMI patients while they are undergoing PCI. Notably, the incidence of adverse cardiac events was considerably higher in patients with low AP levels than in those with high AP levels [95]. Similarly, Ying *et al*. [96] reported that serum AP levels can predict spontaneous reperfusion in STEMI patients.

### 7.2 Apelin in HF

A close relationship has been reported between left ventricular diastolic dysfunction and AP levels. Specifically, plasma AP levels have been found to increase when the left ventricular diastolic function is impaired [97]. Accordingly, a study explored whether serum AP levels were associated with HF with a preserved vote score in T2DM patients. ELISA was used to measure serum N terminal pro-B-type natriuretic peptide (NT-proBNP) and AP levels in all patients. The AP to NT-proBNP ratio was a better predictor of HFpEF in T2DM patients than AP or NT-proBNP alone [98]. Additionally, the prognostic value of AP levels has been investigated in CHF children. Thus, according to the findings, serum AP levels were substantially lower in HF patients than in healthy controls at admission and decreased further with HF progression. Patients with a poorer prognosis had significantly lower serum AP levels than those with a good prognosis. These results suggest that serum AP levels can serve as an effective predictive marker for unfavorable outcomes in children with CHF and HF [99].

### 7.3 Apelin in AS

The relationship between serum AP levels and the severity of calcific aortic stenosis has been investigated. Plasma AP-36 levels were significantly lower in patients with severe AS than in controls and those with mild AS. Furthermore, AP levels were reduced, whereas high-sensitivity C-reactive protein (hsCRP) levels increased in patients with severely calcified AS [100]. cIMT is a well-established tool for AS detection. Several studies have used ELISA to measure serum AP levels in T2DM patients and B-mode ultrasound to assess cIMT. Serum AP and cIMT were positively correlated, which suggested that serum AP is closely related to the degree of carotid AS and offers a promising prognostic biomarker [101]. Moreover, coronary artery ectasia (CAE) is considered a variant of coronary AS. A study analyzed the relationship between serum AP-13 levels and CAE by enrolling 40 CAE patients and determining their serum AP-13 levels. The serum AP-13 levels were significantly lower in the CAE patients than in the CAD patients. The serum AP-13 levels were slightly lower in the CAD group than in the control group [102].

Moreover, various studies have assessed the correlation of serum levels of asymmetric dimethyl arginine, low density lipoprotein receptor-1 (LOX-1), and AP-13 with cIMT; echocardiographic parameters (such as left ventricular mass (LVM) and LVM index (LVMI); and inflammatory markers such as hsCRP and neutrophil: lymphocyte ratio in hemodialysis patients. Compared with the control group, the hemodialysis group exhibited significantly elevated serum AP-13 levels, which were positively correlated with LVM, LVMI, hsCRP, and cIMT [103].

### 7.4 Apelin in Diabetes

The findings suggest that serum AP is potentially linked to carotid AS severity. Meanwhile, type 1 DM (T1DM) is a prevalent chronic disease among children. In a case-control study, serum AP, chemins, cholesterol, TG levels, and albuminuria were all significantly elevated in T1DM patients compared with the controls [104]. In the diabetic group, a significant positive correlation was noted between hemoglobin A1c% (HbA1c%) of AP and chemical proteins, and albuminuria. However, AP was inversely correlated with the glomerular filtration rate [104]. Peripheral neuropathy is a common complication of both T1DM and T2DM. Individuals with diabetic neuropathy exhibited higher plasma AP levels than the healthy controls, and plasma AP exhibited a statistically significant positive correlation with diabetes duration, age, and BMI [105].

Patients with diabetic nephropathy have higher plasma AP levels than healthy subjects and those with T2DM without nephropathy. Additionally, AP levels were positively correlated with disease progression, systolic and diastolic blood pressure, weight, height, fasting blood glucose, 2-hour postprandial glucose, glycosylated hemoglobin, TC, LDL-C, urea, and Cr levels. Conversely, AP levels were negatively correlated with HDL-C and eGFR [106]. Underlying medical conditions such as hypertension, diabetes, and obesity are considered risk factors for the severity of COVID-19 infection. Patients with hypertension and obesity exhibited lower serum AP levels than the control groups. Moreover, AP content was lower in patients with COVID-19 and comorbid diabetes than in their non-COVID-19 counterparts. Serum AP levels were positively correlated with arterial oxygen partial pressure and negatively correlated with the severity of pulmonary involvement [107]. A meta-analysis involving 1493 patients with GDM and 1488 normal pregnant women exhibited no significant differences in circulating AP levels [108]. Nonetheless, another meta-analysis reported significantly elevated AP levels in GDM patients during the second half of pregnancy [109].

## 8. Follistatin-Like 1

FSTL1, also known as TCI-36 and Follistatin-Related Protein (FRP), is a gene induced by the expression of TGF-β1. It was initially identified in osteoblast cell lines [110]. FSTL1 is a stromal cell protein upregulated in various developmental and disease processes, including idiopathic pulmonary fibrosis, keloids, and arthritis. Furthermore, FSTL1 is a cardiac factor highly expressed and released into the serum following cardiac injury. This protein has been associated with CVD and poor prognosis [111]. Thus, whether FSTL1 and its homologs can serve as a useful biomarker for predicting adverse outcomes and death must be investigated. A comprehensive review of this protein family is critical.

### 8.1 FSTL1 in MI

FSTL1 plays a protective role in MI, as it induces cardiac angiogenesis in rats after MI through DIP2A-Smad2/3 signaling [112]. Studies have also measured the plasma FSTL1 levels in AMI patients through ELISA where persistent FSTL1 production in the infarcted myocardium was associated with adverse left ventricle remodeling in AMI survivors [113].

### 8.2 FSTL1 in HF

FSTL1 is an emerging cardiac myokine that is upregulated in HF and has cardioprotective effects in animal cardiac injury models [114]. Similarly, circulating FSTL1 levels were assessed in serum samples of HF patients through ELISAs. FSTL1 levels were elevated in HfpEF patients [115]. In further studies, plasma FSTL1 levels were strongly correlated with clinical parameters in patients who received drug-eluting stents or underwent selective PCI. Specifically, FSTL1 levels were positively correlated with hsCRP, serum Cr, and N-terminal pro-BNP. Elevated FSTL1 levels were identified as an independent predictor of major adverse cardiac and cerebrovascular events, suggesting that FSTL1 levels can serve as a valuable prognostic marker for cardiovascular events in patients receiving elective PCI [116]. Moreover, the potential role of FSTL1 in the context of left ventricular hypertrophy has been highlighted in studies, indicating that elevated serum FSTL1 levels in patients with chronic systolic HF can serve as a reliable marker for left ventricular remodeling [117].

### 8.3 FSTL1 in AS

FSTL1 possesses both pro-inflammatory and anti-inflammatory effects during inflammation. In patients with vasculitis, serum FSTL1 levels were found to be predictive of their current functional status [118]. In another study involving 230 Korean patients, FSTL1 levels were evaluated alongside fully characterized metabolic unhealthy states and coronary plaque presence. The study suggested that fasting venous plasma FSTL1 levels, as measured using ELISA kits, serve as a useful biomarker for metabolic unhealthy status and CVD risk [119].

### 8.4 FSTL1 in Hypertension

Hypertensive patients exhibit lower blood BDNF and myogenin levels, along with increased leptin and irisin levels, than the controls. Likewise, non-obese individuals have reduced concentrations of dickkopf-related protein 1 (DKK1), BDNF, and FSTL1, whereas elevated concentrations of leptin and irisin [120]. Meanwhile, animal studies have focused further on FSTL1. Studies have indicated that FSTL1 can promote tissue remodeling in cases of cardiovascular injury. Patients with COPD-associated pulmonary hypertension and mouse models of hypoxia-induced pulmonary hypertension exhibited elevated serum FSTL1 levels [121].

### 8.5 FSTL1 in Diabetes

FSTL1 is involved in the pathogenesis of obesity and T2DM. Higher levels of FSTL1, Wingless-type inducible signaling pathway protein 1 (WISP1), and asprosin were noted in obese or diabetic patients compared with normal controls, thereby suggesting their potential role in the development of these conditions. Conversely, secreted frizzled-related protein 5, Metrnl, Neuregulin-4, and family with sequence similarity 19 member A5 may serve as protective factors [122]. FSTL1 may ameliorate cardiac dysfunction in MI by inhibiting myocardial fibrosis and apoptosis through upregulation of FSTL1/USP10/Notch1 signaling [123].

Apart from the aforementioned myokines, some other myokines have the potential to serve as biomarkers for CVD. One such myokine is DCN, which is a chondroitin sulfate proteoglycan that interacts with other proteoglycans. DCN plays a role in the MI remodeling process by promoting cardiomyocyte survival following IR injury [124]. Serum DCN levels were significantly higher in patients with acute coronary syndrome (ACS) than in the control group [125]. Notably, serum DCN levels were significantly higher in pre-eclamptic women than in both controls and chronically hypertensive pregnant women [126]. Another critical myokine is myonectin, also known as CTRP15—C1q/TNF-related protein [127]. T2DM patients have a noticeably reduced circulating myonectin level compared with the control group. Additionally, serum myonectin levels were notably lower in the obese non-diabetic control cohort than in the lean non-diabetic control group. In diabetes patients, the serum myonectin concentration was significantly inversely correlated with BMI, as well as other indices [128].

## 9. Conclusions & Future Directions

Accumulating evidence supports the role of certain myokines in CVD, with some having the potential to serve as novel biomarkers reflecting disease development (Table 1, Ref. [12, 13, 14, 18, 19, 20, 21, 24, 25, 26, 34, 38, 39, 40, 41, 44, 46, 47, 54, 56, 57, 60, 62, 63, 65, 66, 67, 72, 73, 77, 78, 81, 82, 83, 87, 88, 89, 90, 91, 95, 96, 97, 99, 100, 101, 102, 103, 104, 105, 106, 107, 108, 113, 115, 116, 117, 119, 120, 121, 122, 125, 126, 128]). Myokines are involved in CVD development and can provide new insights into CVD progression and help identify patients at a high risk of poor prognosis. First, the link between high myostatin levels and an increased risk of CVD has been well established. Similarly, serum irisin levels have been reported to predict cumulative clinical outcomes in patients with HF and T2DM. Furthermore, BDNF, MG53, and AP levels can be used as indicators for the prediction, prognosis, and diagnosis of CVD. Moreover, increasing evidence suggests that FSTL1 is associated with CVD and poor prognosis.

**Table 1.** **Circulating myokines as novel biomarkers for cardiovascular diseases**.

Myokines	Type	Sample Source	Concentrations	Level	References
Myostatin	Serum	Patients with AMI	2375 ng/L	↑	[12]
	Serum	Patients died with MI	<2.20 ng/mL	↓	[13]
	Blood	Mice with MI/ischemia	N/A	↑	[14]
	Serum	Patients with HF	6.5 ± 2.0 ng/mL	↓	[18]
	Serum	Patients with congestive HF	16.28 ± 5.34 ng/mL	↑	[19]
	Serum	Male adolescents with aortic stiffness	0.2 ng/mL	↑	[20]
	Blood	Patients with AS	N/A	↑	[21]
	Plasma	Patients with antihypertensive and lipid-lowering medications	N/A	↓	[26]
	Serum	Obese patients	3702 ± 1384 pg/mL	↑	[25]
	Plasma	Cats with congestive HF	60 ng/mL	↑	[24]
Irisin	Serum	Rat with I/R	<600 ng/mL	↑	[34]
	Serum	Patients with sarcopenia, COPD and CHF	<200 ng/mL	↓	[38]
	Blood	Patients with CHF	2.6 µg/mL	↓	[39]
	Serum	Patients with HFrEF	2.77 ± 0.77 ng/mL	↓	[40]
	Serum	Patients with HFpEF	7.72 ± 0.76 ng/mL	↑	[40]
	Serum	Patients with HFpEF and T2DM	<10.4 ng/mL	↓	[41]
	Serum	Women with preeclampsia	N/A	↓	[44]
	Serum	Patients with T2DM	N/A	↓	[44]
	Serum	Pregnant women with GDM	N/A	↓	[44]
	Serum	Patients with IPAH	≥7.3 µg/mL	↓	[46]
	Serum	Patients with acromegaly	102.71 ± 31.66 ng/mL	↑	[47]
BDNF	Serum	Patients with MI	< 30 ng/mL	↓	[54]
	Serum	Patients with CHF	<25 ng/mL	↓	[56]
	Serum	Patients with HF	N/A	↓	[57]
	Serum	Patients with Chagas cardiomyopathy	≤2.5 ng/mL	↓	[60]
	Serum	Patients with CHF and stroke	N/A	↓	[62]
	Serum	Patients with DMAS	N/A	↓	[63]
	Serum	Patients with T2DM	3854.71 ± 1492.18 pg/mL	↑	[65]
	Serum	Patients with essential hypertension	N/A	↓	[66]
	Serum	Infants of mothers with GDM	328 ± 47 pg/dL	↓	[67]
MG53	Serum	Patients with STEMI	132.17 pg/mL	↑	[72]
	Serum	Patients with stable coronary heart	94.12 ± 48.94 pg/mL	↑	[73]
	Serum	Mice with metabolic syndrome	N/A	↓	[78]
	Serum	Patients with T2DM	120.1 ± 76.7 pg/mL	↑	[77]
Metrnl	Serum	Patients with CVD	132.41 pg/mL	↓	[81]
	Serum	Patients with AMI	≤2.55 ng/mL	↓	[82]
	Serum	Patients with CHF	168.68 pg/mL	↓	[83]
	Plasma	Non-obese women with GDM	941 pg/mL	↓	[87]
	Serum	Patients with diabetic	N/A	↓	[88]
	Serum	Patients with T2DM	1219.9 pg/mL	↑	[89]
	Serum	Obese adult patients	N/A	↓	[90]
	Serum	Patients with T2DM	578.9 ± 225.1 ng/mL	↓	[91]
Apelin	Serum	Patients with STEMI	<0.54 ng/mL	↓	[95]
	Serum	Patients with Spontaneous reperfusion	0.82 ± 0.34 ng/mL	↑	[96]
	Serum	Patients with left ventricular diastolic function	>0.3 ng/mL	↑	[97]
	Serum	Children with HF caused by CHF	126 ng/L	↓	[99]
	Serum	Patients with severe AS	490 pg/mL	↓	[100]
	Serum	Patients with T2DM	407.96 ± 291.07 ng/dL	↑	[101]
	Serum	Patients with CAE	1.86 ± 0.59 ng/mL	↓	[102]
	Serum	Hemodialysis patients	N/A	↑	[103]
	Serum	Patients with T1DM	N/A	↑	[104]
	Serum	Patients with diabetic neuropathy	957.433 ± 221.031 pg/dL	↑	[105]
	Plasma	Patients with diabetic nephropathy	325.79 ± 59.42 pg/mL	↑	[106]
	Serum	Patients with COVID-19 and hypertension/obesity	<100 ng/mL	↓	[107]
	Serum	Pregnancy patients with GDM	N/A	↑	[108]
FSTL1	Plasma	Patients with AMI	N/A	↑	[113]
	Serum	Patients with HF	<400 ng/mL	↑	[115]
	Serum	Patients with drug-eluting stents/PCI	1.41 ng/mL	↑	[116]
	Serum	Patients with chronic systolic HF	N/A	↑	[117]
	Plasma	Patients with Subclinical atherosclerosis	N/A	↑	[119]
	Blood	Patients with hypertension	N/A	↓	[120]
	Serum	Patients with pulmonary hypertension	N/A	↑	[121]
	Serum	Mouse with hypoxia-induced pulmonary hypertension	N/A	↑	[121]
	Serum	Patients with obesity and T2DM	N/A	↑	[122]
DCN	Serum	Patients with ACS	13.59 ± 0.50 pg/mL	↑	[125]
	Serum	Women with preeclampsia	62.33 ng/mL	↑	[126]
Myonectin	Serum	Patients with T2DM	211.87 ± 9.45 ng/mL	↓	[128]

AMI, acute myocardial infarction; ACS, acute coronary syndrome; AS, aortic atherosclerosis; BDNF, brain-derived neurotrophic factor; COPD, chronic obstructive pulmonary disease; CHF, chronic heart failure; CAE, coronary artery ectasia; DMAS, diabetes mellitus induced aortic atherosclerosis; DCN, decorin; FSTL1, follistatin-like 1; GDM, gestational diabetes mellitus; HF, heart failure; HFrEF, HF patients with a reduced ejection fraction; HfpEF, HF patients with a preserved ejection fraction; I/R, ischemia/reperfusion; IPAH, idiopathic pulmonary arterial hypertension; MI, myocardial infarction; MG53, mitsugumin 53; Metrnl, meteorin-like; PCI, percutaneous coronary intervention; STEMI, ST-segment elevation myocardial infarction; T2DM, type 2 diabetes mellitus; T1DM, type 1 diabetes mellitus; CVD, cardiovascular diseases; COVID-19, Corona Virus Disease 2019; N/A, not available.

Although myokines serve as predictive biomarkers independently associated with an increased risk of cardiovascular-related myopathy and cachexia, future studies are warranted to determine their role in predicting adverse cardiac remodeling and risk stratification for clinical outcomes. Additionally, Metrnl, which is a recently discovered secreted protein, is promising as a new biomarker for CHD. Furthermore, the roles of potential biomarkers of CVD, such as DCN and myonectin, in predicting cardiovascular disease and poor prognosis need to be further explored.

Future studies in this field should focus on the following aspects: (1) Efficacy, specificity, sensitivity, and reliability: Comprehensive studies comparing myokines with current biomarkers must be conducted to determine their effectiveness in diagnosing and monitoring CVD. (2) Crosstalk with other cytokines: Further investigation is warranted to understand the interaction between myokines and other cytokines, such as adipokines, cardiokines, and hepatokines, and how this crosstalk impacts CVD development and progression. (3) Influence of exercise: As myokines are secreted by skeletal muscles and can be stimulated by exercise, how exercise affects their performance as biomarkers in CVD must be examined. This will help determine if exercise can influence the accuracy and reliability of myokines as diagnostic and prognostic tools. (4) Myokine cocktail: The significance of a myokine cocktail, which contains more than two myokines, must be determined. Evaluating the presence and interaction of multiple myokines can offer valuable insights into their combined role in CVD pathogenesis. (5) Clinical utility: To fully assess the potential of myokines as clinical biomarkers in CVD, further studies are required to evaluate their diagnostic, prognostic, and therapeutic applications. This will help determine their effectiveness in aiding clinical decision-making and developing targeted therapeutic interventions. In summary, myokines can serve as novel biomarkers for diagnosing, predicting outcomes and developing treatments for CVD.

## Abbreviations

ACS, acute coronary syndrome; AP, Apelin; ACTRII, activin type II receptor; ADMA, asymmetric dimethyl arginine; AH, Arterial hypertension; ALB, albumin; AMI, acute myocardial infarction; Ang II, angiotensin II; AS, atherosclerosis; AT1, angiotensin type 1; BDNF, brain-derived neurotrophic factor; BMI, body mass index; BNP, B-type natriuretic peptide; CAD, coronary artery disease; CAE, coronary artery ectasia; CHF, chronic heart failure; cIMT, carotid intima-media thickness; CKD, chronic kidney disease; COPD, chronic obstructive pulmonary disease; Cr, creatinine; cTnT, cardiac troponin T; CVD, cardiovascular diseases; CHD, coronary heart disease; C-PVAT, close to the proximal aorta; DCN, decorin; DKK1, dickkopf-related protein 1; DM, diabetes mellitus; DMAS, diabetes-induced atherosclerosis; Ecs, Endothelial cells; EFT, epicardial fat thickness; eGFR, estimated glomerular filtration rate; FSTL1, follistatin-like 1; GDF-8, growth differentiation factor 8; GDM, gestational diabetes mellitus; GH, growth hormone; GPx, glutathione peroxidase; HbA1c%, Hemoglobin A1c%; HDL-C, high-density lipoprotein cholesterol; HF, heart failure; HfmrEF, HF with mid-range ejection fraction; HFpEF, Heart failure with preserved ejection fraction; HFrEF, Heart failure with reduced ejection fraction; HOMA-IR, model assessment–insulin; hsCRP, high-sensitivity C-reactive protein; I/R, ischemia/reperfusion; IPAH, idiopathic pulmonary arterial hypertension; IRS-1, insulin receptor substrate 1; IMA-PVAT, internal mammary artery; LVEF, left ventricular ejection fraction; LVM, left ventricular mass; LVMI, left ventricular mass index; LOX-1, low density lipoprotein receptor-1; LDL, low-density lipoprotein; LDL-C, low-density lipoprotein cholesterol; Metrnl, meteorin-like; MG53, mitsugumin 53; MI, myocardial infarction; NGT, normal glucose tolerance; NO, nitric oxide; PCI, percutaneous coronary intervention; PBMCs, peripheral blood mononuclear cells; rhMG53, recombinant human MG53; ROS, reactive oxygen species; sdLDL, small dense low-density lipoprotein; SOD-1, superoxide dismutase -1; STEMI, ST-segment elevation myocardial infarction; T1DM, type 1 diabetes mellitus; T2DM, type 2 diabetes mellitus; TG, triglyceride; TC, total cholesterol; TGF-β, transforming growth factor-β; TRIM, tripartite motif protein; UA, uric acid; UII, urotensin II; VFA, visceral fat accumulation; VFO, visceral adiposity; VSMCs, vascular smooth muscle cells; WISP1, Wingless-type inducible signaling pathway protein 1.
